# Supplementary material for: The decline of the 2022 Italian mpox epidemic: Role of behavior changes and control strategies
Source: Nat Commun. 2024 Mar 13;15:2283. doi: 10.1038/s41467-024-46590-4 (PMC10937928; doi:10.1038/s41467-024-46590-4)
Supplement: Supplementary file 1 — Supplementary Information [file 41467_2024_46590_MOESM1_ESM.pdf]

Supplementary Material

The decline of the 2022 Italian mpox epidemic: role of behavior changes and control strategies

Giorgio Guzzetta [1], Valentina Marziano [1], Alessia Mammone [2], Andrea Siddu [2], Federica Ferraro [2], Anna Caraglia [2], Francesco Maraglino [2], Giovanni Rezza [2,3], Alessandro Vespignani [4], Ira Longini [5], Marco Ajelli [6], Stefano Merler [1,*]

1. Center for Health Emergencies, Bruno Kessler Foundation, Trento, Italy
2. Health Prevention Directorate, Ministry of Health, Rome, Italy
3. Vita-Salute San Raffaele University, Milan, Italy
4. Laboratory for the Modeling of Biological and Socio-Technical Systems, Northeastern University, Boston, MA, USA
5. Department of Biostatistics, Colleges of Public Health and Health Professions, and Medicine, University of Florida, Gainesville, FL, USA
6. Laboratory for Computational Epidemiology and Public Health, Department of Epidemiology and Biostatistics, Indiana University School of Public Health, Bloomington, Indiana, United States

[*] Corresponding author: Stefano Merler - merler@fbk.eu

1. INDIVIDUAL-BASED MODEL OF MPOX TRANSMISSION 2

1.1 THE SEXUALLY ACTIVE MSM POPULATION AND THEIR HOUSEHOLDS 2
1.2 MSM SEXUAL CONTACT NETWORK 2
1.3 CLUB ATTENDANCE 3
1.4 MODEL INITIALIZATION 4
1.5 TRANSMISSION 4
1.6 SELF-REPORTING 5
1.7 CONTACT-TRACING 5
1.8 VACCINATION 6

2. CALIBRATION 8

3. COMPUTATION OF RELEVANT MODEL OUTPUTS 11

3.1 REPRODUCTION NUMBERS 11
3.2 GENERATION TIMES 11

4. SENSITIVITY ANALYSES 12

4.1 ASSORTATIVE MIXING BY SEXUAL ACTIVITY 12
4.2 ASSORTATIVE MIXING BY SEXUAL ACTIVITY AND AGE 16
4.3 MAXIMUM NUMBER OF PARTNERS IN THE SEXUAL CONTACT NETWORK 20
4.4 ATTENDANCE TO CLUBS 23

5. SUPPLEMENTARY REFERENCES 28

1. Individual-Based Model of mpox transmission

We developed an Individual-Based Model (IBM) that accounts for mpox transmission in three settings: households, the sexual contact network among males-who-have-sex-with-males (MSM), and recreational clubs for MSM. Below we provide details on how these settings were implemented.

1.1 The sexually active MSM population and their households

We generated synthetic households by reproducing household sizes and the age relationship between household members reported in Italian census data [1], as described in detail in previous works [2]. In each household, we assigned the status of MSM to household members aged between 15 and 70, based on a Bernoulli sample with probability 5% (corresponding to the hypothesis that 10% of the male Italian population between 15 and 70 years old engages in homosexual activity [3] and that 50% of individuals are women). We considered the age-specific probability of being sexually active, depending on the declared age at sexual debut in data from the Italian national report of the European MSM Internet Survey, version 2017 (EMIS-2017-IT) [4]. We fitted a discrete negative exponential distribution to the probability of experiencing sexual debut by age, in such a way to reproduce the cumulative probability of sexual debut reported in the EMIS-2017-IT report (Figure S1), using the mean squared error to measure the goodness-of-fit. The cumulative probability reported in Figure S1 has the following mathematical expression:

$$P_{sexual\ debut}(a) = \begin{cases} 0 & \text{for } a < 15 \\ 1 - e^{-\phi(a-14)} & \text{for } a \geq 15 \end{cases}$$

with ϕ=0.14 years^{-1}.

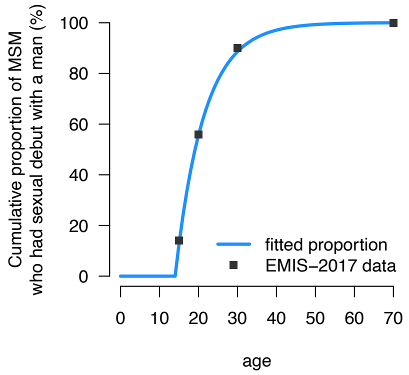

Figure S1. Cumulative probability of sexual debut with a man by age among Italian MSM. Data elaboration from EMIS-2017-IT [4].

After discarding households that did not contain sexually active MSM (as they would not contribute to transmission), we obtained a population of 1.67 million sexually active MSM, and 3.63 million non-MSM household members.

1.2 MSM sexual contact network

According to EMIS-2017-IT data, about 26% of all individuals reported to have had one steady sexual partner in the last 12 months, and the same proportion declared to have had no non-steady sexual partners; therefore, we approximated this fraction to represent individuals in monogamous couples. For the remainder of the sexually active MSM population, we considered non-steady sexual partnerships, according to the proportion of individuals who declared a given number of non-steady yearly partners in EMIS-2017-IT data. Because available data were binned in coarse categories (1, 2, 3-5, 6-10, >10 yearly sexual partners), especially for higher levels of sexual activity, we considered a

discrete probability for non-steady partnership equal to $p(n) = a\,e^{-\omega n}, n \geq 1$ and we fitted the values of a and ω in such a way that to reproduce EMIS-2017-IT data using least squares. The best fit was obtained with a value of a=0.092 and ω=0.117 (Figure S2).

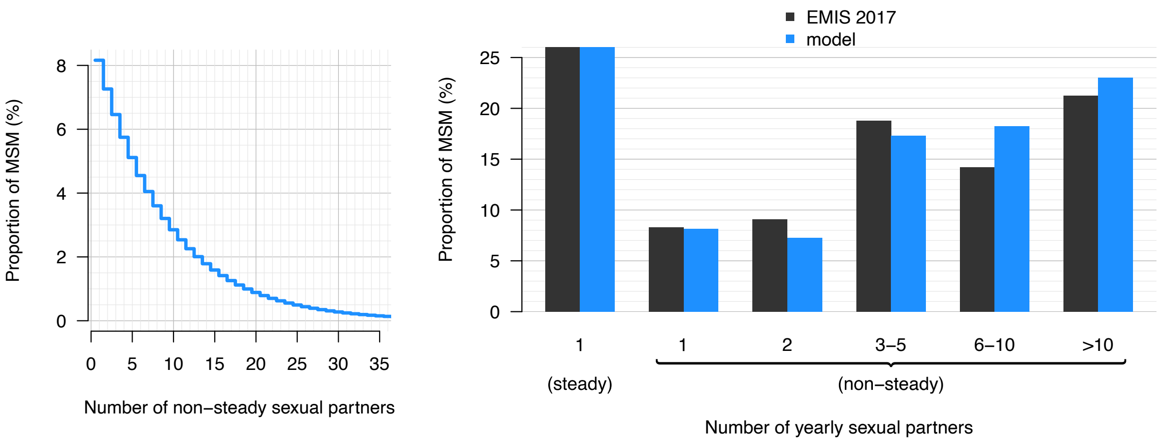

Figure S2. Distribution of the number of non-steady yearly sexual partners. Left: modeled distribution for non-steady sexual partners; right: comparison with corresponding EMIS-2017-IT data [4].

To build the network of sexual partners, we considered 26% of all sexually active MSM to be in monogamous couples. Monogamous couples were built by choosing, for individuals with 1 steady sexual partner, another individual with 1 steady sexual partner. Then, each individual *i* in the remaining 74% was initially assigned a yearly number of non-steady sexual partners, N_i, choosing from the distribution in Figure S2; however, if an individual had more than 30 yearly sexual partners, the partners assigned in the sexual network were exactly 30, and the remaining number of partners was assumed to derive from attendance to MSM recreational clubs (see Section 1.3). The limit on the number of sexual partners in the contact network was removed in a sensitivity analysis (see Section 4.3). The list of non-steady sexual partners for each MSM *i* not in a monogamous couple was assigned iteratively according to the following algorithm:

a. sample a candidate sexual partner *j* randomly from the whole MSM population with probability proportional to their number of yearly sexual partners;
b. if the number of sexual partners already assigned to *j*, K_j, is equal to N_j, reject the candidate and revert to point a.; this rejection criteria was modified in two sensitivity analyses to consider the possibility of assortative mixing by sexual activity and age (see Section 4.1 and 4.2);
c. after finding a suitable candidate, assign *j* to the list of sexual partners of *i*, and *i* to the list of sexual partners of *j*;
d. increase K_i and K_j by 1;
e. if K_i has reached the number of assigned sexual partners N_i, stop the algorithm for *i* and continue with a new individual *r* for which $K_r < N_r$.

Given that the simulation horizon is shorter than one year (from May 9, 2022, to February 28, 2023), the sexual contact network is built once, at the beginning of each simulation, and does not change over time.

1.3 Club attendance

In Italy, all MSM recreational clubs allowing for sex-on-premises are affiliated to a single national association, whose website reported the existence of 50 clubs in 2022[5]. In the model, we generated these 50 clubs by assigning them a maximum capacity assumed to be uniformly distributed between 25 and 275 customers (mean 150), for a total average capacity of approximately 7,500 customers. The clubs were populated one day every week to represent peaks of attendance during the weekend. Given the small size of the potential customer population (about ~0.4% of the total modelled MSM population) and the nature and purpose of such clubs, attendance in the model was restricted to

high-risk (HR) MSM (~2.5% of the total MSM population, see Table 1 in the main text). With this assumption, the club capacity in a given week will be saturated if about 0.4%/2.3% = 17% of HR-MSM attends a club, corresponding to an average attending frequency of once every 6 weeks. In a sensitivity analysis, we allow any MSM in the model to attend a club until saturation capacity (see Section 4.4).
Clubs are sparsely distributed on the Italian territory, therefore customers will generally attend only a few of them for proximity reasons. To reproduce this feature and avoid excessive mixing among the MSM population in clubs, we assigned each MSM a reduced list of *n* clubs (with *n* between 1 and 5). For each individual, the *n* clubs were sampled with probability proportional to their capacity at the beginning of each simulation.

1.4 Model initialization

We initialize the population as fully susceptible and we import one mpox case every day for the first 10 days, in order to simulate the seeding of cases from abroad in the initial phases of the epidemics [6]. Since the temporal evolution of simulated epidemics may be highly stochastic in the early phase, we did not associate a specific date to the first importations, but we aligned simulated epidemic trajectories in such a way that the 58th diagnosed case would occur on June 8, 2022 (as in actual data), designating the end of Period 1. From that time point on, simulation time will follow calendar time with Periods 2, 3, and 4 lasting 30 days and Period 5 lasting until the end of simulations on February 28, 2023.

1.5 Transmission

At any time-step, an infectious individual can transmit to other individuals through the modeled routes of infection:

- within households, an infectious individual can transmit to any susceptible household member (independently of whether this happens via fomites or close contacts, including sexual ones), according to a probability given by $1 - e^{\frac{\beta_H}{N_H}\Delta t}$, where β_H is the mpox transmissibility parameter within households (free model parameter), N_H is the total number of household members and Δt is the time-step of the simulation.
- In the MSM sexual contact network, transmission can occur only between an infectious individual and sexual partners in their list, according to a probability given by $1 - e^{\beta_S \chi_S(t)\, \Delta t}$, where β_S is the mpox transmissibility parameter in the MSM sexual contact network (free model parameter) and $\chi_S(t)$ is a parameter that distinguishes the level of transmissibility before and after the end of Period 1:
$$\chi_S(t) = \begin{cases} 1 & \text{for } t \text{ in Period 1.} \\ 1 - \chi_S & \text{for } t \text{ in Periods } 2-5 \end{cases}$$
with $0 \le \chi_S \le 1$ being the relative reduction of transmissibility in the MSM sexual contact network after Period 1 (free model parameter).
In absence of data, the model does not account for preferential sexual contacts with individuals within the list of contacts (i.e., it cannot distinguish between repeated encounters with a non-steady sexual partner and occasional sexual intercourse).
- In MSM recreational clubs, an attending infectious individual can transmit to any of the other MSM attending the same club at the same time, according to a probability $1 - e^{\frac{\beta_C}{N_C}\chi_C(t)\, \Delta t}$, where β_C is the mpox transmissibility parameter in MSM recreational clubs (free model parameter), N_C is the total club population for that week, and $\chi_C(t)$ is a parameter that distinguishes the level of transmissibility in clubs before and after the end of Period 1:
$$\chi_C(t) = \begin{cases} 1 & \text{for } t \text{ in Period 1.} \\ 1 - \chi_C & \text{for } t \text{ in Periods } 2-5 \end{cases}$$
with $0 \le \chi_C \le 1$ being the relative reduction of transmissibility in clubs after Period 1 (free model parameter).

In the model, infectious non-MSM individuals can transmit only through the household transmission route.
The transmissibility parameters related to sexual transmission in the contact network β_S and in clubs β_C can be interpreted as the combination of two components: the frequency of sexual intercourse and the probability of mpox transmission per sexual intercourse. The relative reductions of transmissibility in the sexual network and in clubs, χ_S and χ_C, can be interpreted as the effect of spontaneous behavior change on both components, e.g., the potential reduction in the frequency of intercourse in the sexual network and in clubs, or the adoption of safer sex practices affecting the probability of mpox transmission per sexual intercourse. There are no reliable data to estimate the frequency of sexual intercourse in the sexual network and in clubs in Italy, and the estimated probability of mpox transmission per sexual intercourse is highly variable in the different studies [7–9]; the relative reductions in both components following behavior change are also unknown. For this reason, the estimated transmissibility parameters and their reductions refer to the infection transmission process and cannot distinguish its individual determinants.

1.6 Self-reporting

We assume that all infectious individuals remain exposed and do not transmit until symptom development, which occurs after an incubation period sampled from a distribution estimated from Italian data[6]. At the date of symptom development, a Bernoulli sample with probability equal to the self-reporting probability ζ (free model parameter) will define whether the individual will be diagnosed in the future or will remain unnoticed by the surveillance system. In the first case, the date at which self-reporting will occur is established by adding to the date of symptom onset a delay (in days) sampled from empirical distributions. In particular, we fitted discretized gamma-functions to observed data from two different phases of the epidemic (Figure S3): Period 1, during which awareness of mpox symptoms was less widespread among cases, and therefore self-presentation was slightly more delayed (average 8.2 days); and Periods 2-5, where shorter diagnostic delays have been observed (average 6.5 days). After diagnosis of a case, we assume instantaneous and perfect isolation. Undiagnosed individuals will continue to transmit until the end of their infectious period, assumed to be exponentially distributed with average 20 days [10].

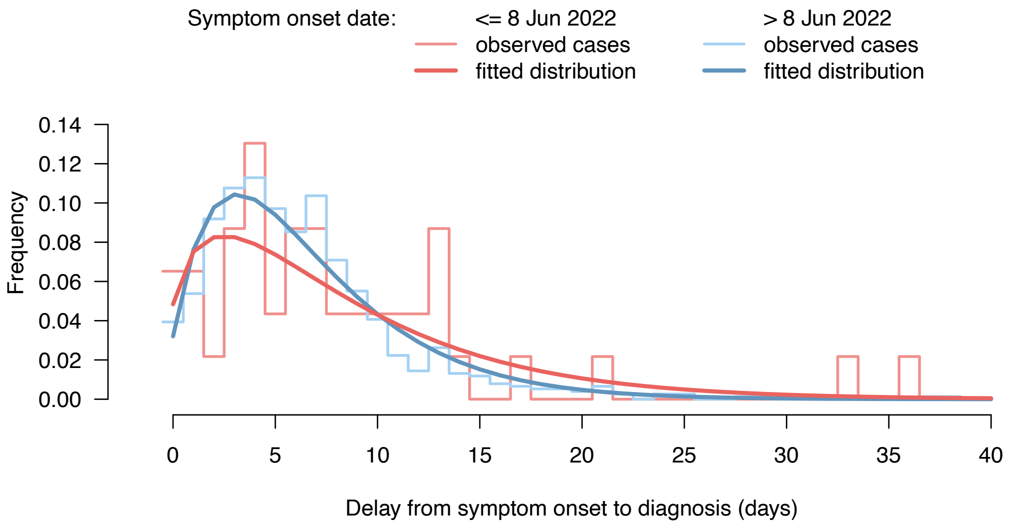

Figure S3. Diagnostic delays for cases with symptom onset before and after June 8, 2022. Data from Italian Ministry of Health.

1.7 Contact-tracing

Upon diagnosis of a case, contact tracing is initiated. We assume that all household members of a case and a fraction of their contacts in the sexual network are traced and (if infected) diagnosed,

between 1 and 3 days from the diagnosis of the index case, including the test turnaround time. We assume that contacts within clubs are not traced due to their occasional nature. The fraction of sexual contacts traced, also referred to as contact tracing coverage, was set in the baseline analysis to 20%. This value allowed to reproduce available data on the proportion of diagnosed cases who were known contacts of a previously diagnosed case (see Figure 1D in the main text). In counterfactual scenarios, we assume different contact tracing coverage values, that traced contacts may be additionally vaccinated (see Section 1.8), and/or that contacts of contacts may also be traced and tested (ring tracing) and vaccinated (ring vaccination). Figure S4 below shows that choosing values for the contact tracing coverage different than 20% results in an incorrect proportion of diagnosed cases who were known contacts of a previously diagnosed case, thereby validating our choice for the baseline analysis.

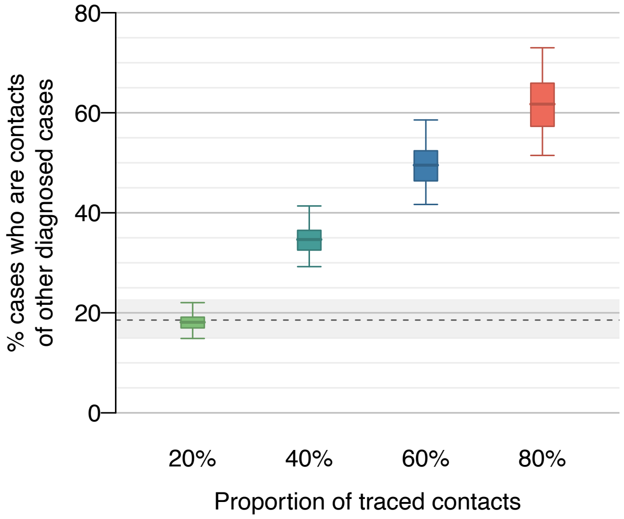

Figure S4. Percentage of cases diagnosed via contact tracing under different contact tracing coverage values. Boxplots represents the mean (central bar), interquartile range (IQR, rectangular box) and 95% prediction intervals (PI) (whiskers); the gray horizontal dashed line and shaded area represent the mean and 95% confidence interval (CI) of the binomial distribution for the probability that a diagnosed case is a contact of another case in observed data.

1.8 Vaccination

In all scenarios considered (including the baseline scenario), an immunization campaign was simulated to reproduce the one administered by regional health authorities under the coordination of the Ministry of Health since August 8, 2022, prioritizing MSM with high sexual activity. Data on the number of first doses administered per day (Figure S5) were used to determine the daily number of HR-MSM to be vaccinated in the model. We assumed that all individuals who received a first dose will automatically receive their second dose after a delay of 28 days from the first. In alternative scenarios, vaccine is additionally administered to traced contacts of cases and to contacts of contacts (ring vaccination).

We assume that vaccination administered to infected individuals or individuals with a previous mpox infection will not have any effect. Vaccination on susceptible individuals provides a partial protection against infection $\rho(t)$ that changes over time according to the following equation:

$$\rho(t) = \begin{cases} 0 & \text{for } t < t_v + \tau_{r1} \\ \rho_1 & \text{for } t_v + \tau_{r1} \le t < t_v + \tau_{d2} + \tau_{r2} \\ \rho_2 & \text{for } t \ge t_v + \tau_{d2} + \tau_{r2} \end{cases}$$

Where ρ_1 and ρ_2 are the effectiveness associated to the first and second dose of vaccine, t_v is the time at which the first dose is administered, τ_{r1} and τ_{r2} are the ramp-up times after the first and second dose, and τ_{d2} is the delay between the first and second dose. The values adopted are

reported in Table S1. Vaccinated individuals have a reduced probability of being infected upon contact at time t with an infectious individual, with relative risk equal to 1- $\rho(t)$.

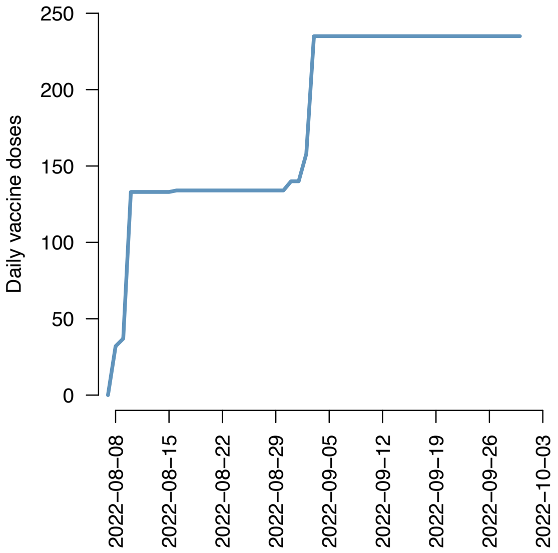

Figure S5. Rollout of the vaccination campaign prioritized on HR-MSM individuals. Data from the Italian Ministry of Health.

Table S1. Parameters for vaccine effectiveness and ramp-up.

SYMBOL	PARAMETER	VALUE	SOURCE
$\boldsymbol{\rho_1}$	Effectiveness after first dose	80%	[11]
$\boldsymbol{\rho_2}$	Effectiveness after second dose	90%	[12,13]
$\boldsymbol{\tau_{r1}}$	Ramp-up time after first dose	14 days	[11]
$\boldsymbol{\tau_{r2}}$	Ramp-up time after second dose	14 days	[14]
$\boldsymbol{\tau_{d2}}$	Delay between first and second dose	28 days	Italian Ministry of Health

2. Calibration

Free model parameters were:

- the mpox transmissibility in households, β_H,
- the mpox transmissibility in the MSM sexual contact network, β_S,
- the mpox transmissibility in MSM recreational clubs, β_C;
- the relative reduction in transmissibility in the sexual contact network after June 8, 2022, χ_S,
- the relative reduction in transmissibility in clubs after June 8, 2022, χ_C,
- the probability of self-reporting, ζ.

We used an Approximate Bayesian Computation based on Sequential Monte Carlo (ABC-SMC) to calibrate the model, with two sequential steps.
In Step 1, we sampled 100,000 parameter values independently and uniformly from the range of values reported in Table S2. For the three transmissibility parameters, we explored values broad enough to allow the distributions of selected parameters to be fully contained within the selected range. For all parameters represented by a percentage (χ_S, χ_C, and ζ), we explored the full range from 0 to 100%.

Table S2. Range of explored parameter values

SYMBOL	PARAMETER	MIN	MAX
$\boldsymbol{\beta_H}$	Transmissibility in households	0.00	0.30
$\boldsymbol{\beta_S}$	Transmissibility in the sexual contact network	0.00	30.00
$\boldsymbol{\beta_C}$	Transmissibility in clubs	0.00	0.075
$\boldsymbol{\chi_S}$	Relative reduction of transmissibility in the sexual contact network	0%	100%
$\boldsymbol{\chi_C}$	Relative reduction of transmissibility in clubs	0%	100%
$\boldsymbol{\zeta}$	Probability of self-reporting	0%	100%

Each parameter set was run with one stochastic repetition and its performance was evaluated by considering the following log-likelihood as a score function:

$$LL(\boldsymbol{D}|\boldsymbol{M}(\boldsymbol{\theta})) = \left(\prod_{1}^{4} Pois(D_i, M_i(\boldsymbol{\theta}))\right) \cdot Multinom(\boldsymbol{Q_1}, \boldsymbol{Z_1}(\boldsymbol{\theta})) \cdot Multinom(\boldsymbol{Q_2}, \boldsymbol{Z_2}(\boldsymbol{\theta}))$$

where:

- $\boldsymbol{D}$ represents the overall dataset, $\boldsymbol{D} = \{D_1, D_2, D_3, D_4, \boldsymbol{Q_1}, \boldsymbol{Q_2}\}$;
  - D_i represents the observed total number of diagnosed cases with symptom onset in Period *i*;
  - $\boldsymbol{Q_i} = \{Q_{a,i}, Q_{b,i}, Q_{c,i}, Q_{d,i}\}$ represents the number of observed cases in Period *i* associated to categories defined by epidemiological investigations:
    a. cases who had another confirmed case in the household (we refer to this category as “Household”);
    b. cases who did not have another case confirmed in the household and had attended a sauna, sex club, party or LGBT pride event in the three weeks preceding symptom onset (we refer to this category as “Club”);
    c. cases who did not have another case confirmed in the household and had not attended a club in the preceding three weeks, and who reported ≤2 sexual partners in the three weeks preceding symptom onset (we refer to this category as “Sex, low activity”);
    d. cases who did not have another case confirmed in the household and had not attended a club in the preceding three weeks, and who reported ≥ 3 sexual partners in the three weeks preceding symptom onset (we refer to this category as “Sex, high activity”);

- $\boldsymbol{M}(\boldsymbol{\theta})$ represents model outputs, $\boldsymbol{M} = \{M_1, M_2, M_3, M_4, \boldsymbol{Z_1}, \boldsymbol{Z_2}\}$, obtained with a given set of parameter values, $\boldsymbol{\theta} = \{\beta_H, \beta_S, \beta_C, \chi_S, \chi_C, \zeta\}$;
    - M_i represents the modeled total number of diagnosed cases with symptom onset in Period *i*;
    - $\boldsymbol{Z_i} = \{Z_{a,i}, Z_{b,i}, Z_{c,i}, Z_{d,i}\}$ represents the fraction of cases being associated to corresponding categories during Period *i*, within model simulations, using the same criteria as in actual epidemiological investigation.
- $Pois(k, \lambda)$ represents the Poisson likelihood of observing k cases given a mean rate of λ
- $Multinom(\boldsymbol{A}, \boldsymbol{p})$ represent the multinomial likelihood of finding $\boldsymbol{A} = \{A_a, A_b, A_c, A_d)$ cases, respectively, in categories $a, b, c,$ and d given an underlying set of probabilities $\boldsymbol{p} = \{p_a, p_b, p_c, p_d\}$, with $\sum_i p_i = 1$

For fitting data on the total number of diagnosed cases, four periods were considered (Period 1 to Period 4). For fitting data on categorized cases, only Periods 1 and 2 were considered, because starting from Period 3 epidemiological information were sufficient to assign only a small fraction of cases to specific categories of likely exposure (<15%, as opposed to >50% in periods 1 and 2).

We selected parameter sets $\widetilde{\boldsymbol{\theta}}_1$ associated to simulations for which the resulting log-likelihood was within three times the marginal log-likelihood obtained with a perfect fit of the data:

$$\widetilde{\boldsymbol{\theta}}_1 : LL\left(\boldsymbol{D}\middle|\widetilde{\boldsymbol{\theta}}_1\right) \leq 3 \cdot LL(\boldsymbol{D}|\boldsymbol{D})$$

In Step 2, we sampled 5,000 parameters with replacement from the joint distribution $\widetilde{\boldsymbol{\theta}}_1$ and applied to each sample a gaussian kernel perturbation; 20 stochastic repetitions were run for each perturbed parameter, for a total of 100,000 additional simulations. We selected simulations $\widetilde{\boldsymbol{S}}_2$ for which the resulting log-likelihood was within twice the marginal log-likelihood obtained with a perfect fit of the data.

$$\widetilde{\boldsymbol{S}}_2 : LL\left(\boldsymbol{D}\middle|\widetilde{\boldsymbol{S}}_2\right) \leq 2 \cdot LL(\boldsymbol{D}|\boldsymbol{D})$$

We obtained 694 simulations $\widetilde{\boldsymbol{S}}_2$ corresponding to 488 unique parameter sets $\widetilde{\boldsymbol{\theta}}_2$, which constitute our approximation of the joint posterior distribution. Marginal posterior distributions for the free model parameters, weighted by the number of accepted simulations per parameter set, are reported in Figure 2 in the main text and in Figure S6 below.

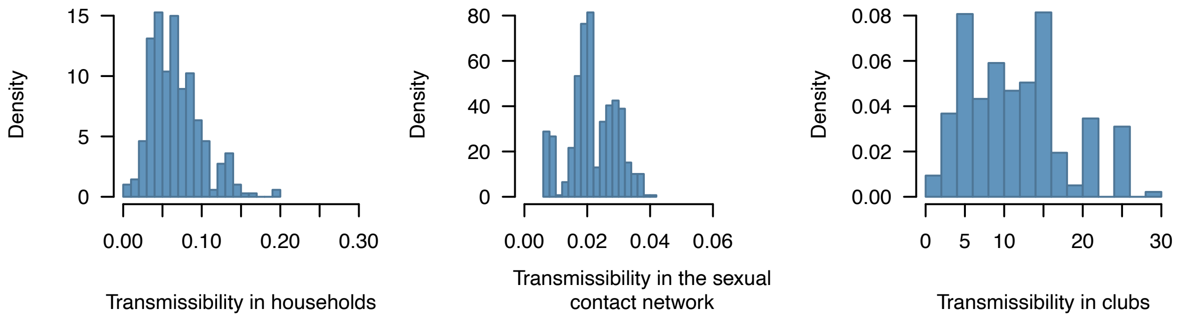

Figure S6. Approximate posterior distribution of transmissibility parameters in households (β_H), in the sexual contact network (β_S) and in clubs (β_C), after calibration.

Figure S7 reports the histogram of the Probability Integral Transform (PIT) values for the ensemble of epidemic trajectories by date of symptom onset (Figure 1c) in the 694 accepted simulations against observed values. The model's PIT values were reasonably approximating a uniform distribution, suggesting an adequate calibration of the model [15]. The residual discrepancies between modeled epidemic trajectories and data may be attributable to the simplification of behavior change as happening all at once rather than gradually.

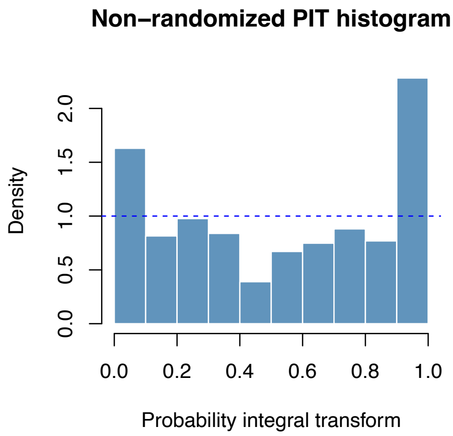

Figure S7. Histogram of Probability Integral Transform values for marginal predictions of daily cases. The dashed horizontal line represents the target uniform distribution.

3. Computation of relevant model outputs

3.1 Reproduction numbers

To compute the average reproduction number in heterogeneous settings, we applied the definition of average number of secondary cases caused by individuals who participate to transmission in that setting, leveraging the IBM's capability of tracking the number of infections caused in each setting by each infected individual. More specifically, we computed:

$$R_X(P) = \frac{c_{2X}(P)}{c_{1,X}(P)}$$

Where

- $R_X(P)$ is the reproduction number in setting $X = \{S, C, H\}$ during period P, with S representing the sexual contact network, C representing clubs and H representing households;
- $c_{1,X}(P)$ is the total number of cases infected within period P who participate in setting X; in particular, $c_{1,S}(P)$ is the number of cases among sexually active MSM; $c_{1,C}(P)$ is the number of cases among HR-MSM, who are allowed to attend clubs; and $c_{1,H}(P)$ is the number of cases among individuals who do not live alone (i.e., who have at least another household member);
- $c_{2,X}(P)$ is the sum of cases transmitted within setting X by individuals counted in $c_{1,X}(P)$.

3.2 Generation times

By tracking the time of infection of primary and their secondary cases, we can estimate a distribution of realized generation times among diagnosed cases, reproducing the estimation performed on contact tracing data in Italy [6]. Assuming that all generation times of secondary cases are perfectly identified, we found a mean modeled generation time of 14.4 days (95% PI: 14.3, 14.6) and a 95-percentile interval of the mean distribution of 5 and 32 days respectively. Previous estimates for Italy suggested a mean of 12.5 days and a 95-percentile interval of 5 and 23 days respectively [6]. Figure S8 shows the good agreement between the modeled generation time distribution and the one computed using a gamma distribution with mean parameters estimated from data [6].

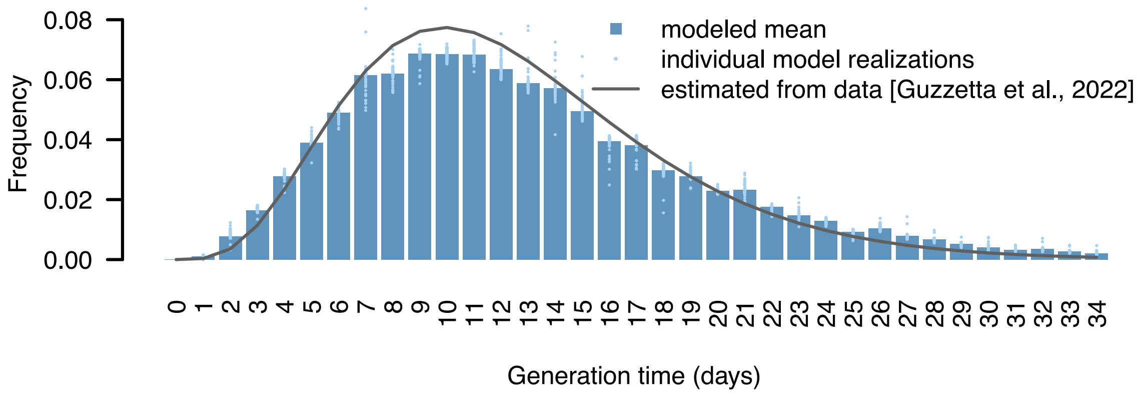

Figure S8. Modeled distribution of the realized generation times across simulations accepted during calibration and distribution obtained from the mean parameters estimated from Italian contact tracing data [6].

4. Sensitivity analyses

To assess the robustness of our conclusions with respect to several model assumptions, we run four sensitivity analyses where we recalibrated the model using the same technique and criteria reported in Section 2 after changing specific details in model structure. We report below the methodology and results of the sensitivity analyses, which generally confirm the conclusions of the main analysis.

4.1 Assortative mixing by sexual activity

In this sensitivity analysis, we assumed that the sexual contact network could be assortative by sexual activity levels, i.e., that MSM have a higher probability to have sexual encounters with individuals with a similar number of yearly sexual partners. In order to implement assortative mixing by sexual activity, we added a further rejection condition in the algorithm for the creation of the non-monogamous component of the sexual network (see Section 1.2) as follows:

a. sample a candidate sexual partner *j* randomly from the whole MSM population with probability proportional to their number of yearly sexual partners;

b. if the number of sexual partners already assigned to *j*, K_j, is equal to N_j, reject the candidate and revert to point a.;

b2. reject the candidate with probability $p_{sex-assortative} = 1 - e^{-\eta|N_i - N_j|}$ and in such case revert to point a.;

c. after finding a suitable candidate, assign *j* to the list of sexual partners of *i*, and *i* to the list of sexual partners of *j*;

d. increase K_i and K_j by 1;

e. if K_i has reached the number of assigned sexual contacts N_i, stop the algorithm for *i* and continue with a new individual *r* for which $K_r < N_r$.

Adding the additional condition **b2** for candidate partners implies that candidates will be always accepted if they have the same number of yearly sexual partners as *i* and will be rejected with higher probability if the difference between N_i and N_j, in whatever direction, is larger. In absence of data, we assumed η= 0.067, obtaining a contact network with characteristics reported in Table S3.

Table S3. Assortativity in the modeled sexual contact networks, excluding monogamous couples and partners encountered in clubs.

SEXUAL ACTIVITY GROUP (YEARLY SEXUAL PARTNERS)	AVERAGE NUMBER OF NON-STEADY YEARLY SEXUAL PARTNERS AMONG PARTNERS
VERY LOW RISK (1-3)	10.9
LOW RISK (4-10)	12.0
MODERATE RISK (11-29)	16.4
HIGH RISK (30+)	21.2

We recalibrated the model using the same method described above, obtaining 604 accepted simulations corresponding to 461 parameter sets representing the joint posterior distributions. Below, we report results presented by pooling together 50 stochastic replicates for each of 100 samples without replacement from the joint posterior distribution of parameters (total 5,000 simulations for each scenario). Figures S9-S14 show results that are qualitatively and quantitatively comparable (except for minor differences) to results presented in the main analysis. In particular, Figure S9 shows that the model with an assortative contact network by sexual activity level fits the data similarly well to the main analysis. Figure S10 reports the posterior distribution of parameter values, which remain similar to the main analysis (self-reporting: 80% with 95% confidence intervals (CI) 33-100%; reduction in sexual contacts in clubs: 81% with 95%CI 33-100%; reduction in sexual contacts in network: 71% with 95%CI 32-87%). Figure S11 shows similar estimates for the total and setting-specific reproduction numbers, similar proportions of infections by setting of transmission and

similar cumulative incidence levels by sexual activity groups. The mean cumulative incidence in HR-MSM (30+ yearly sexual partners) is higher compared to the baseline, as expected for an assortative network (4.2 per 1,000 against 2.5 per 1,000 in the random network), but is still very far from any effect of susceptible depletion. Figures S12-S14 confirm results for alternative intervention scenarios, including preventive immunization of highest-risk individuals.

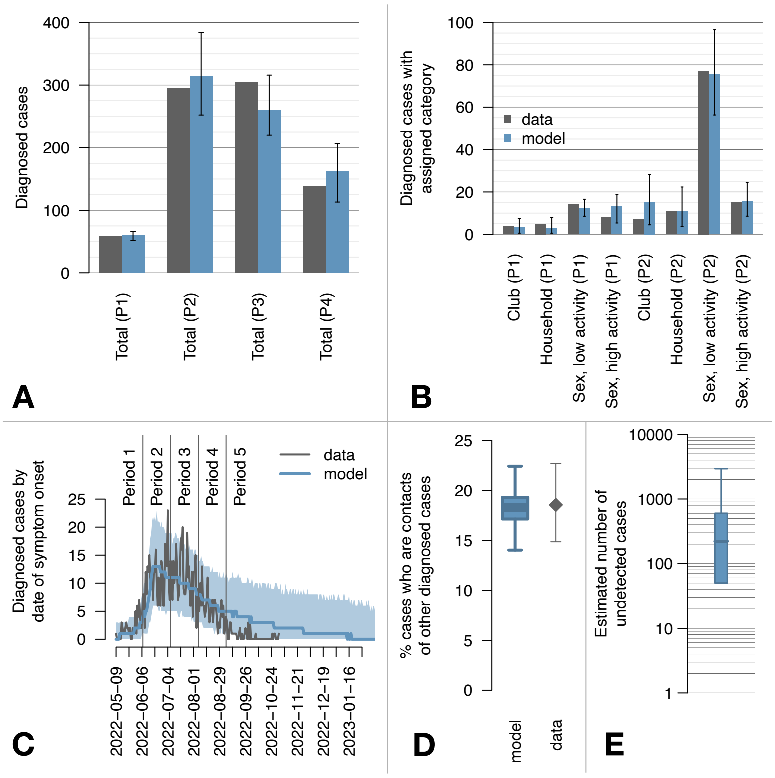

Figure S9. Model fit and validation under the assumption of assortative mixing by sexual activity. A) Total number of diagnosed cases with symptom onset in four epidemiological periods (P1: May 9 – June 7, 2022; P2: June 8 – July 7; P3: July 8 – August 6; P4: August 7 – September 5). Bars represent the observed values (dark gray) and the mean modelled estimates (blue); vertical bars represent 95% prediction intervals (PI). B) Classification of cases in periods P1 and P2 for the subset of cases for which a categorization was available. Categories represent the potential setting of exposure, with "low activity" and "high activity" representing individuals with 2 or less and 3 or more sexual partners disclosed in the preceding 3 weeks, respectively (see Methods). Bars represent the observed values (dark gray) and the mean modelled estimates (blue); vertical bars represent 95% PI. C) Epidemic curve by symptom onset date. Observed data (dark gray line) are compared against the mean modeled curve (blue line) and the 95% PI. D) Percentage of cases diagnosed via contact tracing; the gray diamond and whiskers represent the mean and 95% confidence interval (CI) of the binomial distribution for the probability that a diagnosed case is a contact of another case in observed data. E) Number of undetected mpox cases estimated by the model (y-axis in a log scale); central bar: median; box: interquartile range (IQR); whiskers: 90%PI. In all figures, model variability derives from 604 simulations accepted during calibration.

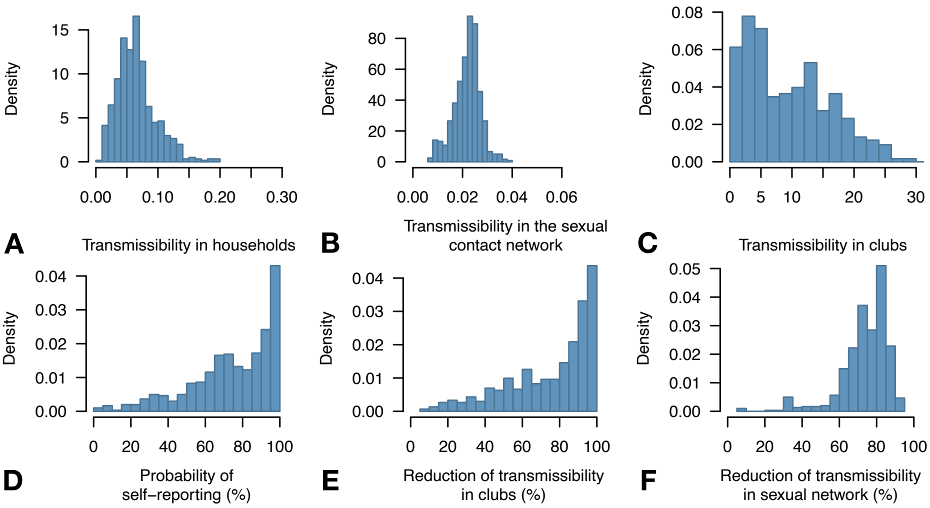

Figure S10. Posterior distributions of parameters under the assumption of assortative mixing by sexual activity. A) Transmissibility in households (β_H). B) Transmissibility in clubs (β_C). C) Transmissibility in the sexual contact network (β_S). D) Proportion of mpox infections that self-report symptoms to the surveillance system and are confirmed as mpox cases (ζ). E) Reduction of the transmission rate in clubs due to spontaneous behavior change, assumed to be in place since June 8, 2022 (χ_C). F) Reduction of the transmission rate in the sexual contact networks due to spontaneous behavior change, assumed to be in place since June 8, 2022 (χ_S).

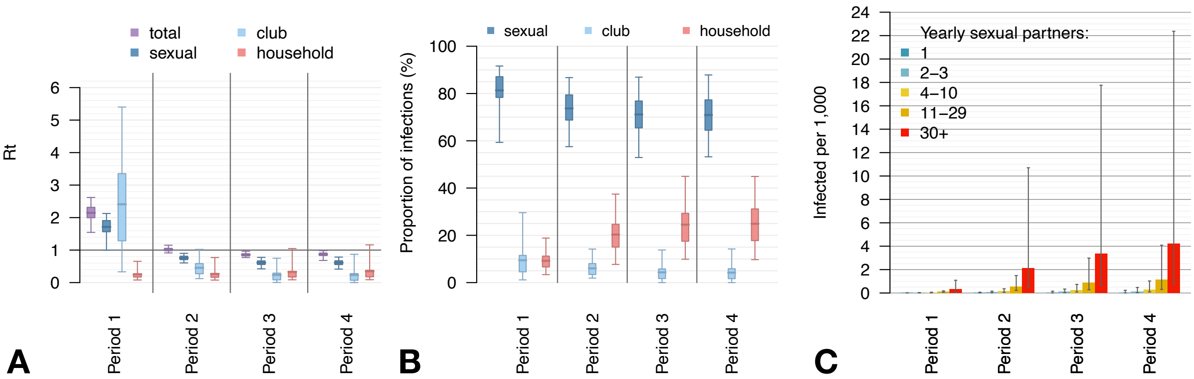

Figure S11. Estimates of mpox transmission dynamics parameters under the assumption of assortative mixing by sexual activity. A) Reproduction number by route of acquisition and period (Period 1: May 9 – June 7, 2022; Period 2: June 8 – July 7; Period 3: July 8 – August 6; Period 4: August 7 – September 5). Boxplots represent mean values (central bars), interquartile ranges (rectangular boxes), and 95%PI (whiskers). B) Proportion of infections by route of acquisition and period. Boxplots represent mean values (central bars), interquartile ranges (rectangular boxes), and 95%PI (whiskers). C) Cumulative incidence rate by number of yearly sexual partners and period. Bars represent mean values and whiskers represent 95%PI.

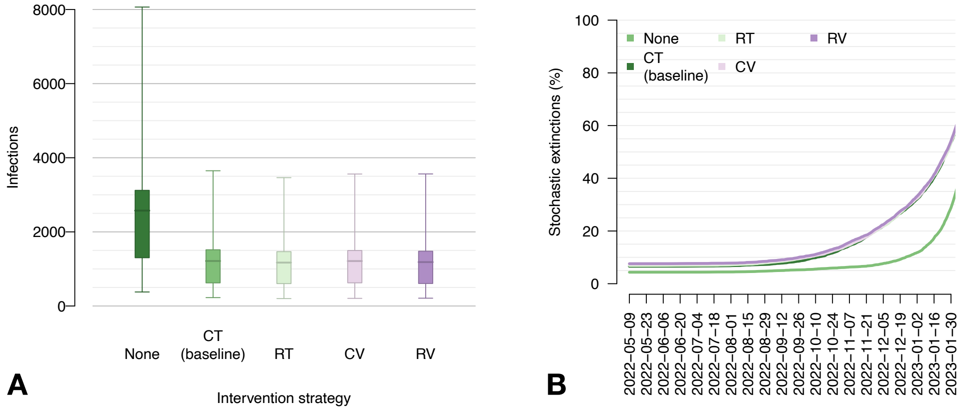

Figure S12. Alternative intervention scenarios under the assumption of assortative mixing by sexual activity. A) Total number of infections across the simulation period (May 9, 2022 – February 28, 2023), by intervention. None: no active intervention; CT: simple contact tracing; RT: ring tracing; CV: vaccination of contacts; RV: ring vaccination. Boxplots represent mean values (central bars), interquartile ranges (rectangular boxes), and 95%PI (whiskers). B) Cumulative percentage of simulations that result in a stochastic extinction over time, by type of intervention. A stochastic extinction occurs when there are no more infectious or exposed individuals in the population.

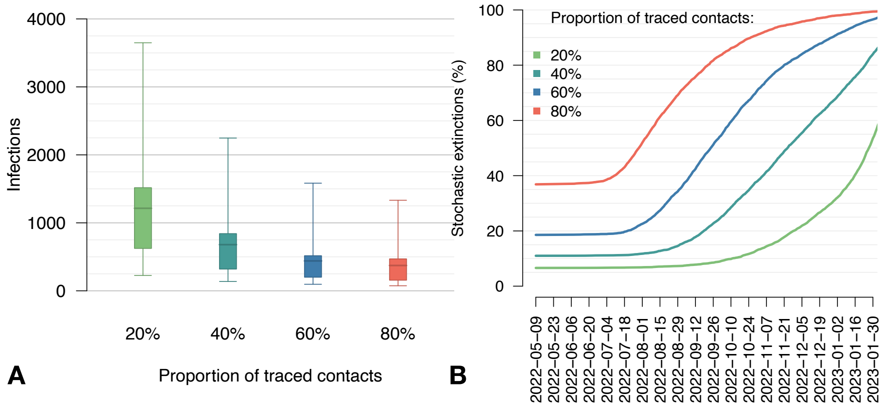

Figure S13. Alternative proportions of sexual contacts that are successfully traced under the assumption of assortative mixing by sexual activity. A) Total number of infections across the simulation period (May 9, 2022 – February 28, 2023), by proportion of traced contacts. None: no active intervention; CT: simple contact tracing; RT: ring tracing; CV: vaccination of contacts; RV: ring vaccination. Boxplots represent mean values (central bars), interquartile ranges (rectangular boxes), and 95%PI (whiskers). B) Cumulative percentage of simulations that result in a stochastic extinction over time, by proportion of traced contacts. A stochastic extinction occurs when there are no more infectious or exposed individuals in the population.

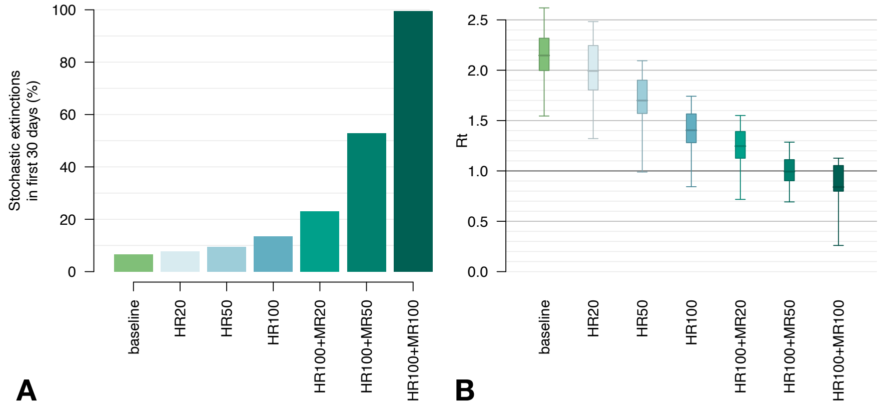

Figure S14. Effect of preventive vaccination on a potential new mpox outbreak under the assumption of assortative mixing by sexual activity. A) Percentage of simulations that end up in a stochastic extinction within 30 days since the first case, by vaccination coverage. A stochastic extinction occurs when there are no more infectious or exposed individuals in the population. HR20: vaccinating 20% of high-risk (HR) MSM, i.e., those with 30 sexual partners per year or more; HR50: vaccinating 50% of HR MSM; HR100: vaccinating all HR MSM; MR20: vaccinating 20% of moderate risk (MR) MSM, i.e., those with 11 to 29 sexual partners per year; MR50: vaccinating 50% of MR MSM; MR100: vaccinating all MR MSM. Boxplots represent mean values (central bars), interquartile ranges (rectangular boxes), and 95%PI (whiskers). B) Reproduction number by vaccination coverage.

4.2 Assortative mixing by sexual activity and age

In this sensitivity analysis, we assumed that the sexual contact network could be assortative by both sexual activity levels and age. We further modified the algorithm for the creation of the non-monogamous component of the sexual network as follows:

a. sample a candidate sexual partner *j* randomly from the whole MSM population with probability proportional to their number of yearly sexual partners;

b. if the number of sexual partners already assigned to *j*, K_j, is equal to N_j, reject the candidate and revert to point a.;

b2. reject the candidate with probability $p_{sex-assortative} = 1 - e^{-\eta|N_i - N_j|}$ and in such case revert to point a.;

b3. reject the candidate with probability $p_{age-assortative} = 1 - e^{-\xi|a_i - a_j|}$, with a_i and a_j being the ages of *i* and *j*, and in such case revert to point a.;

c. after finding a suitable candidate, assign *j* to the list of sexual partners of *i* and *i* to the list of sexual partners of *j*;

d. increase K_i and K_j by 1;

e. if K_i has reached the number of assigned sexual contacts N_i, stop the algorithm for *i* and continue with a new individual *r* for which $K_r < N_r$.

Adding the additional condition **b3** for candidate partners with respect to the sensitivity analysis in Section 4.1 implies that candidates will be always accepted if they have the same age as *i* and will be rejected with higher probability if the difference between their ages, in whatever direction, is larger. In absence of data, we assumed ξ= 0.15, obtaining a relative probability of acceptance of a candidate as in Table S4.

Table S4. Assortativity by age in the modeled sexual contact networks, excluding partners encountered in clubs.

AGE DIFFERENCE (YEARS)	RELATIVE PROBABILITY OF PARTNERSHIP
0	1.00
5	0.472
10	0.223
20	0.050
40	0.002

We recalibrated the model using the same method described above, obtaining 1416 accepted simulations corresponding to 1018 parameter sets representing the joint posterior distributions. Below, we report results presented by pooling together 50 stochastic replicates for each of 100 samples without replacement from the joint posterior distribution of parameters (total 5,000 simulations for each scenario). Figures S15-S20 show results that are qualitatively and quantitatively comparable (except for minor differences) to results presented in the main analysis. In particular, Figure S15 shows that the model with an assortative contact network by sexual activity and age fits the data similarly well to the main analysis. Figure S16 reports the posterior distribution of parameter values, which remain similar to the main analysis (self-reporting: 81% with 95% confidence intervals (CI) 26-95%; reduction in sexual contacts in clubs: 87% with 95%CI 62-99%; reduction in sexual contacts in network: 66% with 95%CI 23-87%). Figure S17 shows similar estimates for the total and setting-specific reproduction numbers, similar proportions of infections by setting of transmission and similar cumulative incidence levels by sexual activity groups. More specifically, the mean cumulative incidence in very high-risk individuals (30+ yearly sexual partners) is higher compared to the baseline, as expected for an assortative network (4.0 per 1,000 against 2.5 per 1,000 in the random network), but is still very far from any effect of susceptible depletion. Figures S18-S20 confirm results for alternative intervention scenarios, including preventive immunization of highest-risk individuals.

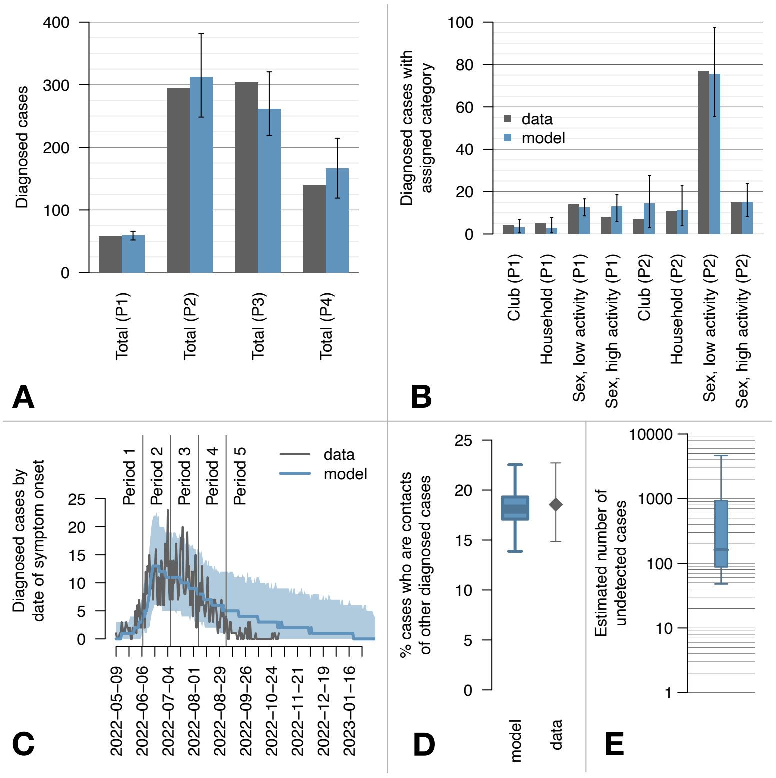

Figure S15. Model fit and validation under the assumption of assortative mixing by sexual activity and age. A) Total number of diagnosed cases with symptom onset in four epidemiological periods (P1: May 9 – June 7, 2022; P2: June 8 – July 7; P3: July 8 – August 6; P4: August 7 – September 5). Bars represent the observed values

(dark gray) and the mean modelled estimates (blue); vertical bars represent 95% prediction intervals (PI). B) Classification of cases in periods P1 and P2 for the subset of cases for which a categorization was available. Categories represent the potential setting of exposure, with "low activity" and "high activity" representing individuals with 2 or less and 3 or more sexual partners disclosed in the preceding 3 weeks, respectively (see Methods). Bars represent the observed values (dark gray) and the mean modelled estimates (blue); vertical bars represent 95% PI. C) Epidemic curve by symptom onset date. Observed data (dark gray line) are compared against the mean modeled curve (blue line) and the 95% PI. D) Percentage of cases diagnosed via contact tracing; the gray diamond and whiskers represent the mean and 95% confidence interval (CI) of the binomial distribution for the probability that a diagnosed case is a contact of another case in observed data. E) Number of undetected mpox cases estimated by the model (y-axis in a log scale); central bar: median; box: interquartile range (IQR); whiskers: 90%PI. In all figures, model variability derives from 604 simulations accepted during calibration.

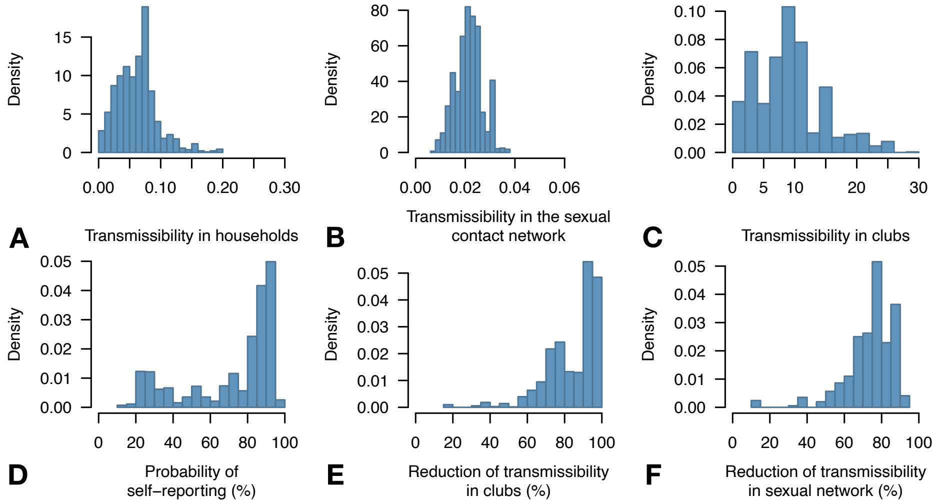

Figure S16. Posterior distributions of parameters under the assumption of assortative mixing by sexual activity and age. A) Transmissibility in households (β_H). B) Transmissibility in clubs (β_C). C) Transmissibility in the sexual contact network (β_S). D) Proportion of mpox infections that self-report symptoms to the surveillance system and are confirmed as mpox cases (ζ). E) Reduction of the transmission rate in clubs due to spontaneous behavior change, assumed to be in place since June 8, 2022 (χ_C). F) Reduction of the transmission rate in the sexual contact networks due to spontaneous behavior change, assumed to be in place since June 8, 2022 (χ_S).

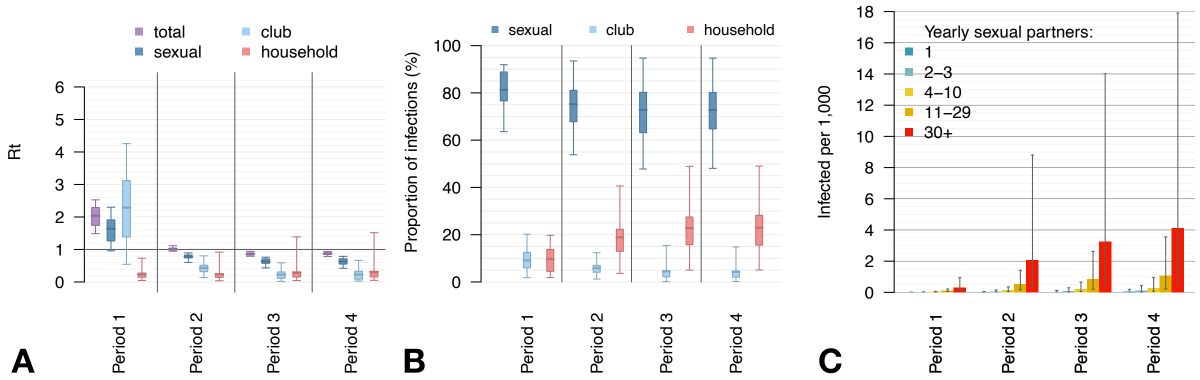

Figure S17. Estimates of mpox transmission dynamics parameters under the assumption of assortative mixing by sexual activity and age. A) Reproduction number by route of acquisition and period (Period 1: May 9 – June 7, 2022; Period 2: June 8 – July 7; Period 3: July 8 – August 6; Period 4: August 7 – September 5). Boxplots represent mean values (central bars), interquartile ranges (rectangular boxes), and 95%PI (whiskers). B) Proportion of infections by route of acquisition and period. Boxplots represent mean values (central bars), interquartile ranges (rectangular boxes), and 95%PI (whiskers). C) Cumulative incidence rate by number of yearly sexual partners and period. Bars represent mean values and whiskers represent 95%PI.

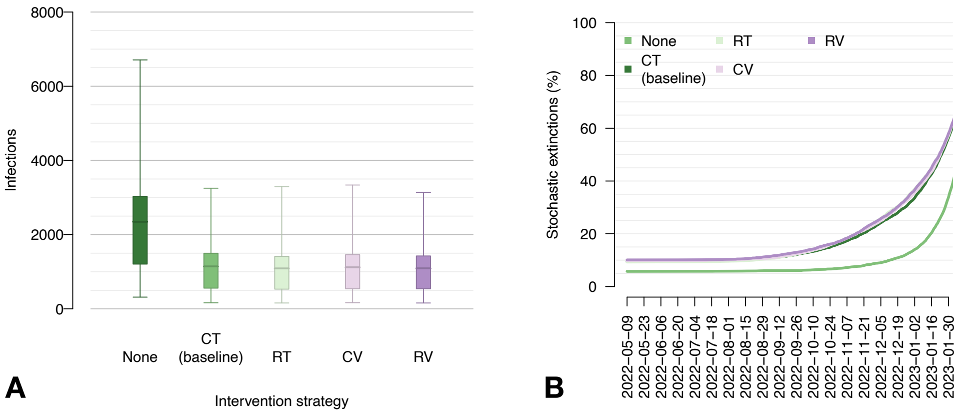

Figure S18. Alternative intervention scenarios under the assumption of assortative mixing by sexual activity and age. A) Total number of infections across the simulation period (May 9, 2022 – February 28, 2023), by intervention. None: no active intervention; CT: simple contact tracing; RT: ring tracing; CV: vaccination of contacts; RV: ring vaccination. Boxplots represent mean values (central bars), interquartile ranges (rectangular boxes), and 95%PI (whiskers). B) Cumulative percentage of simulations that result in a stochastic extinction over time, by type of intervention. A stochastic extinction occurs when there are no more infectious or exposed individuals in the population.

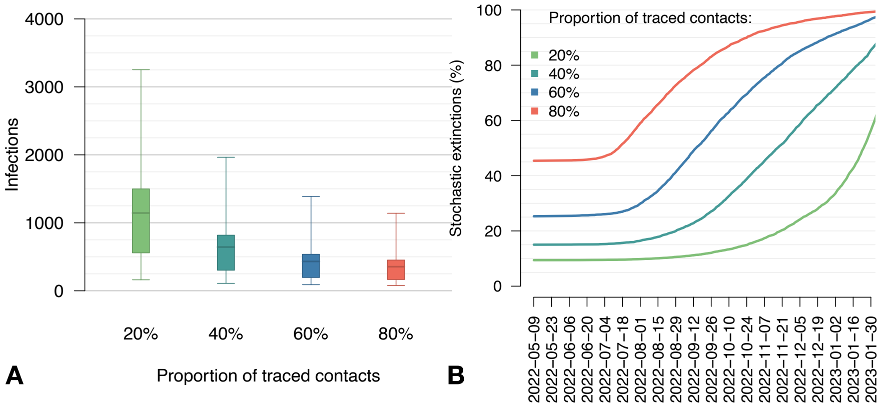

Figure S19. Alternative proportions of sexual contacts that are successfully traced under the assumption of assortative mixing by sexual activity and age. A) Total number of infections across the simulation period (May 9, 2022 – February 28, 2023), by proportion of traced contacts. None: no active intervention; CT: simple contact tracing; RT: ring tracing; CV: vaccination of contacts; RV: ring vaccination. Boxplots represent mean values (central bars), interquartile ranges (rectangular boxes), and 95%PI (whiskers). B) Cumulative percentage of simulations that result in a stochastic extinction over time, by proportion of traced contacts. A stochastic extinction occurs when there are no more infectious or exposed individuals in the population.

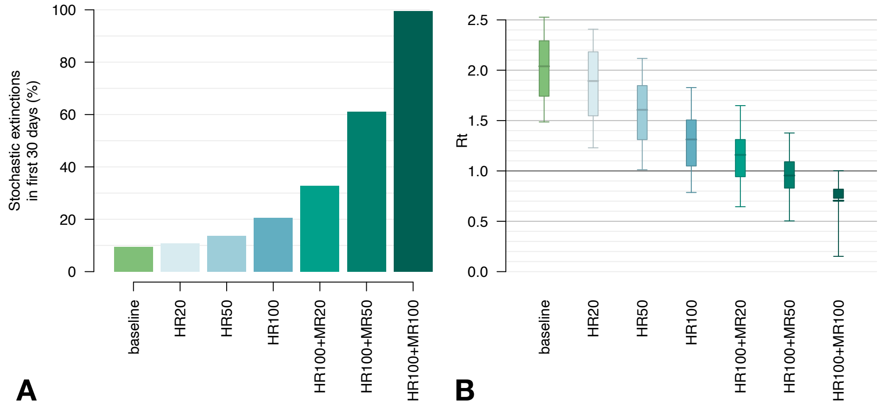

Figure S20. Effect of preventive vaccination on a potential new mpox outbreak under the assumption of assortative mixing by sexual activity and age. A) Percentage of simulations that end up in a stochastic extinction within 30 days since the first case, by vaccination coverage. A stochastic extinction occurs when there are no more infectious or exposed individuals in the population. HR20: vaccinating 20% of high-risk (HR) MSM, i.e., those with 30 sexual partners per year or more; HR50: vaccinating 50% of HR MSM; HR100: vaccinating all HR MSM; MR20: vaccinating 20% of moderate risk (MR) MSM, i.e., those with 11 to 29 sexual partners per year; MR50: vaccinating 50% of MR MSM; MR100: vaccinating all MR MSM. Boxplots represent mean values (central bars), interquartile ranges (rectangular boxes), and 95%PI (whiskers). B) Reproduction number by vaccination coverage.

4.3 Maximum number of partners in the sexual contact network

In this sensitivity analysis, we removed the maximum number of partners in the sexual contact network, which is set to 30 in the baseline analysis. Here, the number of non-steady yearly sexual partners is assigned according to the distribution estimated from data (see Figure S2; note that although the figure is cropped to a maximum value of 35, the actual distribution insists over the interval [0, +∞)). The condition that only MSM with 30+ yearly sexual partners are allowed to attend clubs is maintained. This model likely overestimates the number of yearly sexual partners since additional partnerships are formed through attendance to clubs.

We recalibrated the model using the same method described above, obtaining 1287 accepted simulations corresponding to 987 parameter sets representing the joint posterior distributions. Below, we report results presented by pooling together 50 stochastic replicates for each of 100 samples without replacement from the joint posterior distribution of parameters (total 5,000 simulations for each scenario). Figures S21-S26 show results that are qualitatively and quantitatively comparable (except for minor differences) to results presented in the main analysis. In particular, Figure S21 shows that the model with no maximum number of yearly partners in the sexual contact network fits the data similarly well to the main analysis. Figure S22 reports the posterior distribution of parameter values, which remain similar to the main analysis (self-reporting: 85% with 95% confidence intervals (CI) 42-95%; reduction in sexual contacts in clubs: 82% with 95%CI 63-97%; reduction in sexual contacts in network: 66% with 95%CI 58-83%). Figure S23 shows similar estimates for the total and setting-specific reproduction numbers, similar proportions of infections by setting of transmission and similar cumulative incidence levels by sexual activity groups. Figures S24-S26 confirm results for alternative intervention scenarios, including preventive immunization of highest-risk individuals.

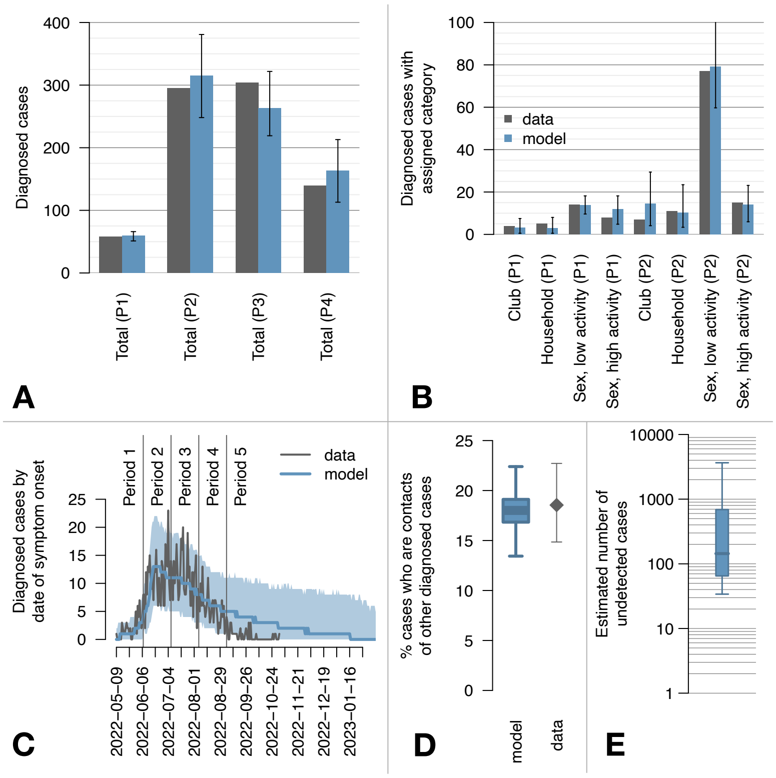

Figure S21. Model fit and validation after removing the limit on the number of yearly partners in the sexual contact network. A) Total number of diagnosed cases with symptom onset in four epidemiological periods (P1: May 9 – June 7, 2022; P2: June 8 – July 7; P3: July 8 – August 6; P4: August 7 – September 5). Bars represent the observed values (dark gray) and the mean modelled estimates (blue); vertical bars represent 95% prediction intervals (PI). B) Classification of cases in periods P1 and P2 for the subset of cases for which a categorization was available. Categories represent the potential setting of exposure, with "low activity" and "high activity" representing individuals with 2 or less and 3 or more sexual partners disclosed in the preceding 3 weeks, respectively (see Methods). Bars represent the observed values (dark gray) and the mean modelled estimates (blue); vertical bars represent 95% PI. C) Epidemic curve by symptom onset date. Observed data (dark gray line) are compared against the mean modeled curve (blue line) and the 95% PI. D) Percentage of cases diagnosed via contact tracing; the gray diamond and whiskers represent the mean and 95% confidence interval (CI) of the binomial distribution for the probability that a diagnosed case is a contact of another case in observed data. E) Number of undetected mpox cases estimated by the model (y-axis in a log scale); central bar: median; box: interquartile range (IQR); whiskers: 90%PI. In all figures, model variability derives from 604 simulations accepted during calibration.

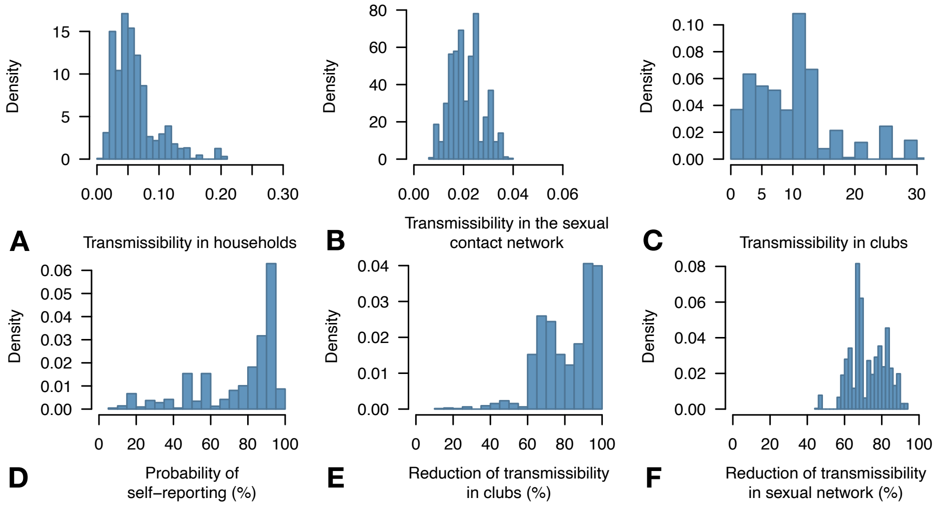

Figure S22. Posterior distributions of parameters after removing the limit on the number of yearly partners in the sexual contact network. A) Transmissibility in households (β_H). B) Transmissibility in clubs (β_C). C) Transmissibility in the sexual contact network (β_S). D) Proportion of mpox infections that self-report symptoms

to the surveillance system and are confirmed as mpox cases (ζ). E) Reduction of the transmission rate in clubs due to spontaneous behavior change, assumed to be in place since June 8, 2022 (χ_C). F) Reduction of the transmission rate in the sexual contact networks due to spontaneous behavior change, assumed to be in place since June 8, 2022 (χ_S).

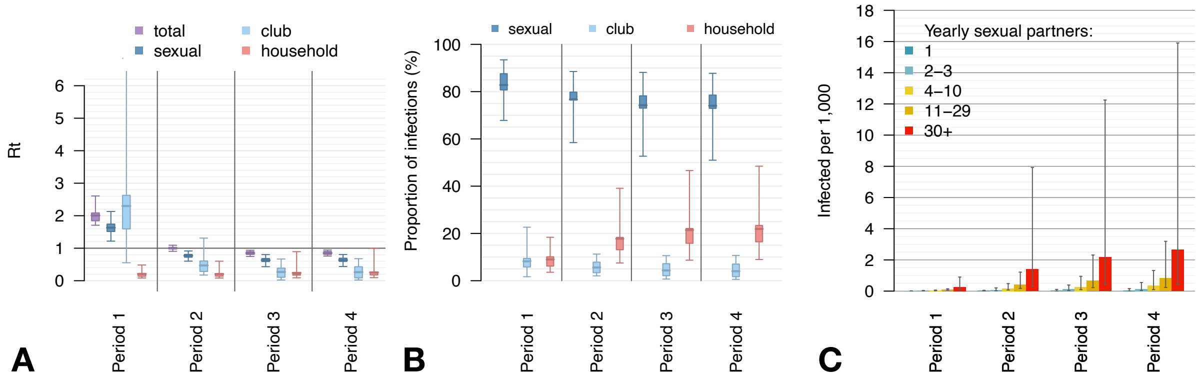

Figure S23. Estimates of mpox transmission dynamics parameters after removing the limit on the number of yearly partners in the sexual contact network. A) Reproduction number by route of acquisition and period (Period 1: May 9 – June 7, 2022; Period 2: June 8 – July 7; Period 3: July 8 – August 6; Period 4: August 7 – September 5). Boxplots represent mean values (central bars), interquartile ranges (rectangular boxes), and 95%PI (whiskers). B) Proportion of infections by route of acquisition and period. Boxplots represent mean values (central bars), interquartile ranges (rectangular boxes), and 95%PI (whiskers). C) Cumulative incidence rate by number of yearly sexual partners and period. Bars represent mean values and whiskers represent 95%PI.

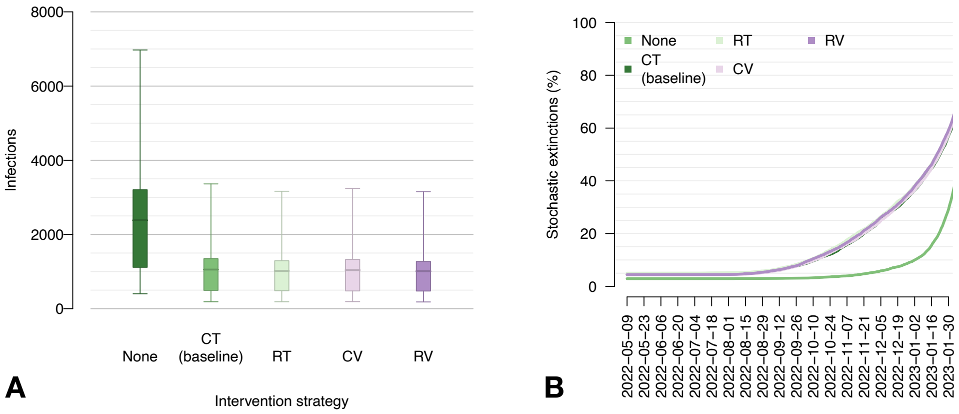

Figure S24. Alternative intervention scenarios after removing the limit on the number of yearly partners in the sexual contact network. A) Total number of infections across the simulation period (May 9, 2022 – February 28, 2023), by intervention. None: no active intervention; CT: simple contact tracing; RT: ring tracing; CV: vaccination of contacts; RV: ring vaccination. Boxplots represent mean values (central bars), interquartile ranges (rectangular boxes), and 95%PI (whiskers). B) Cumulative percentage of simulations that result in a stochastic extinction over time, by type of intervention. A stochastic extinction occurs when there are no more infectious or exposed individuals in the population.

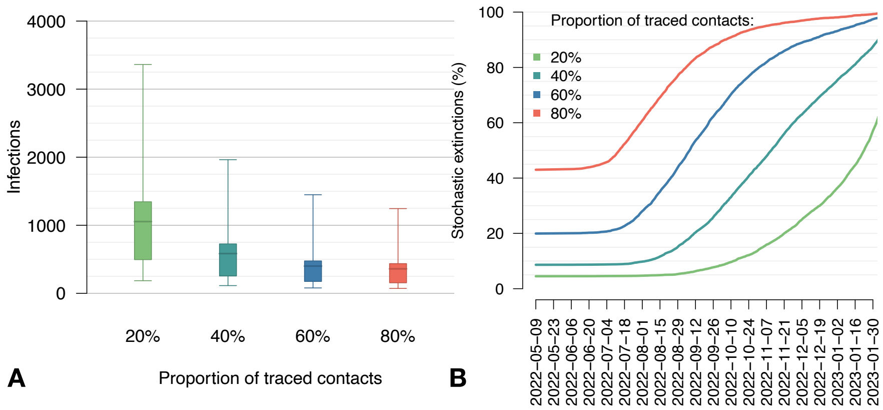

Figure S25. Alternative proportions of sexual contacts that are successfully traced after removing the limit on the number of yearly partners in the sexual contact network. A) Total number of infections across the simulation period (May 9, 2022 – February 28, 2023), by proportion of traced contacts. None: no active intervention; CT: simple contact tracing; RT: ring tracing; CV: vaccination of contacts; RV: ring vaccination. Boxplots represent mean values (central bars), interquartile ranges (rectangular boxes), and 95%PI (whiskers). B) Cumulative percentage of simulations that result in a stochastic extinction over time, by proportion of traced contacts. A stochastic extinction occurs when there are no more infectious or exposed individuals in the population.

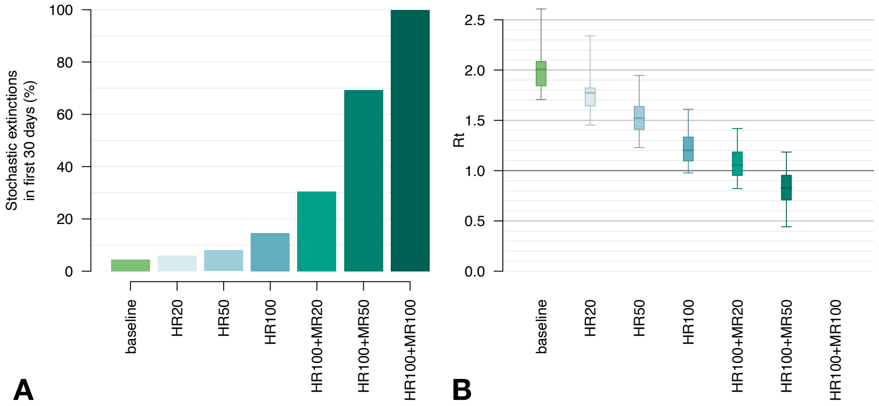

Figure S26. Effect of preventive vaccination on a potential new mpox outbreak after removing the limit on the number of yearly partners in the sexual contact network. A) Percentage of simulations that end up in a stochastic extinction within 30 days since the first case, by vaccination coverage. A stochastic extinction occurs when there are no more infectious or exposed individuals in the population. HR20: vaccinating 20% of high-risk (HR) MSM, i.e., those with 30 sexual partners per year or more; HR50: vaccinating 50% of HR MSM; HR100: vaccinating all HR MSM; MR20: vaccinating 20% of moderate risk (MR) MSM, i.e., those with 11 to 29 sexual partners per year; MR50: vaccinating 50% of MR MSM; MR100: vaccinating all MR MSM. Boxplots represent mean values (central bars), interquartile ranges (rectangular boxes), and 95%PI (whiskers). B) Reproduction number by vaccination coverage.

4.4 Attendance to clubs

In this sensitivity analysis, we changed the baseline model by removing the condition that only MSM with 30+ yearly sexual partners are allowed to attend clubs: any MSM is allowed to attend until saturation of club capacity. This model likely underestimates the proportion of individuals with low sexual activity since a significant number of additional partnerships may be formed through attendance to clubs also by individuals who declare a small number of yearly sexual partners.
We recalibrated the model using the same method described above, obtaining 312 accepted simulations corresponding to 226 parameter sets representing the joint posterior distributions.
Below, we report results presented by pooling together 50 stochastic replicates for each of 100

samples without replacement from the joint posterior distribution of parameters (total 5,000 simulations for each scenario). Figure S27 shows that the model where anyone can attend clubs fits the data similarly well to the main analysis. Figure S28 reports the posterior distribution of parameter values; this model estimates a slightly smaller proportion of self-reporting (67% with 95% confidence intervals (CI) 31-79%), a much smaller reduction of transmissibility in clubs: 36% with 95%CI 15-89%, and a more consistent reduction of transmissibility in the sexual contact network: 78% with 95%CI 70-85%). Compared to the main analysis, a much lower reproduction number in clubs was estimated by the model (mean of about 1.3 against about 2.7 in the main analysis) and a lower proportion of cases transmitted in clubs (about 2.5% against 10% in the main analysis), as shown in Figure S29. The lower importance of club transmission explains why the model can allow a smaller reduction of transmissibility in clubs and requires a higher reduction of transmissibility in the sexual contact network. A higher attack rate was estimated in lower sexual activity groups and a lower attack rate in the higher sexual activity groups, further supporting the conclusion that population immunity in high-risk groups did not contribute significantly to the downturn of the epidemics. Despite differences in the estimated transmission dynamics, Figures S30-S32 generally confirm results for alternative intervention scenarios, except for preventive immunization of highest-risk individuals: in this case, only a scenario where all high- and moderate- risk individuals are vaccinated guarantees a significant proportion of outbreaks to be controlled at the source.

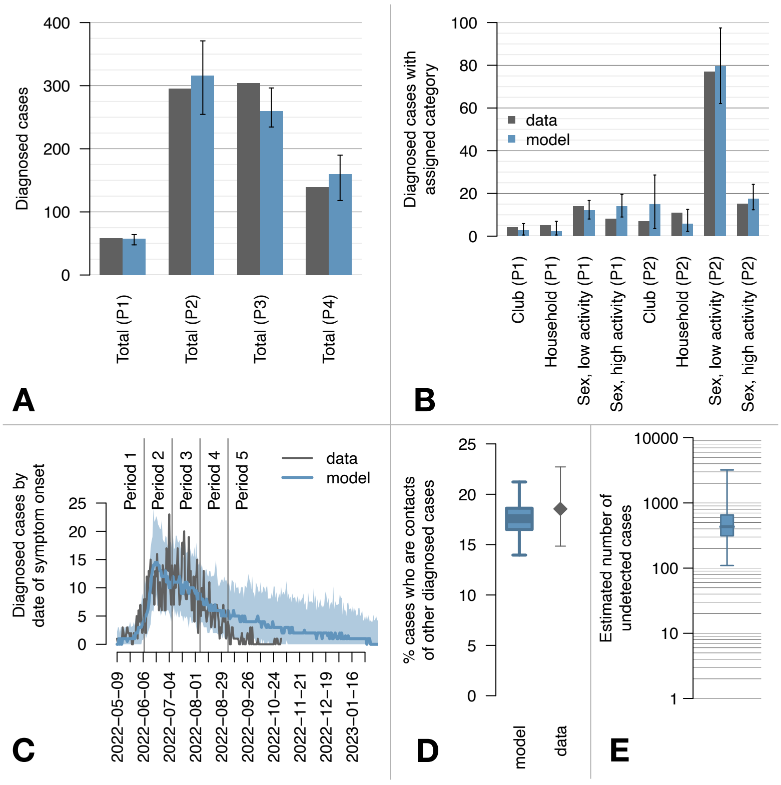

Figure S27. Model fit and validation under the assumption that clubs are attended by MSM irrespectively of their sexual activity level. A) Total number of diagnosed cases with symptom onset in four epidemiological periods (P1: May 9 – June 7, 2022; P2: June 8 – July 7; P3: July 8 – August 6; P4: August 7 – September 5). Bars represent the observed values (dark gray) and the mean modelled estimates (blue); vertical bars represent 95% prediction intervals (PI). B) Classification of cases in periods P1 and P2 for the subset of cases for which a categorization was available. Categories represent the potential setting of exposure, with “low activity” and “high activity” representing individuals with 2 or less and 3 or more sexual partners disclosed in the preceding 3 weeks, respectively (see Methods). Bars represent the observed values (dark gray) and the mean modelled estimates (blue); vertical bars represent 95% PI. C) Epidemic curve by symptom onset date. Observed data (dark gray line) are compared against the mean modeled curve (blue line) and the 95% PI. D) Percentage of cases diagnosed via contact tracing; the gray diamond and whiskers represent the mean and 95% confidence interval (CI) of the binomial distribution for the probability that a diagnosed case is a contact of another case in

observed data. E) Number of undetected mpox cases estimated by the model (y-axis in a log scale); central bar: median; box: interquartile range (IQR); whiskers: 90%PI. In all figures, model variability derives from 604 simulations accepted during calibration.

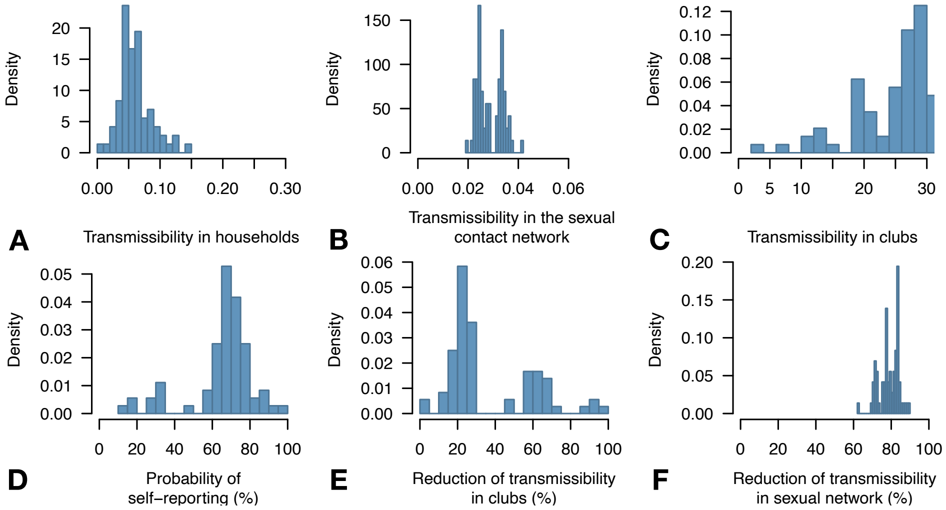

Figure S28. Posterior distributions of parameters under the assumption that clubs are attended by MSM irrespectively of their sexual activity level. A) Transmissibility in households (β_H). B) Transmissibility in clubs (β_C). C) Transmissibility in the sexual contact network (β_S). D) Proportion of mpox infections that self-report symptoms to the surveillance system and are confirmed as mpox cases (ζ). E) Reduction of the transmission rate in clubs due to spontaneous behavior change, assumed to be in place since June 8, 2022 (χ_C). F) Reduction of the transmission rate in the sexual contact networks due to spontaneous behavior change, assumed to be in place since June 8, 2022 (χ_S).

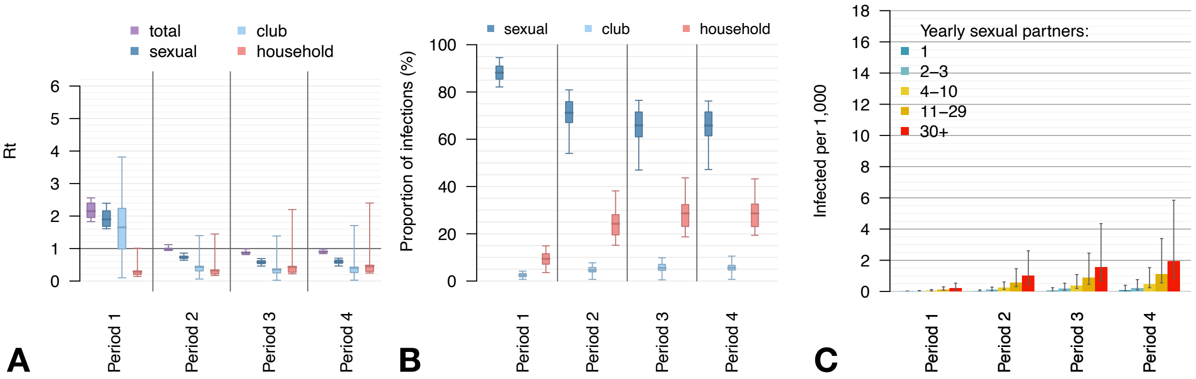

Figure S29. Estimates of mpox transmission dynamics parameters under the assumption that clubs are attended by MSM irrespectively of their sexual activity level. A) Reproduction number by route of acquisition and period (Period 1: May 9 – June 7, 2022; Period 2: June 8 – July 7; Period 3: July 8 – August 6; Period 4: August 7 – September 5). Boxplots represent mean values (central bars), interquartile ranges (rectangular boxes), and 95%PI (whiskers). B) Proportion of infections by route of acquisition and period. Boxplots represent mean values (central bars), interquartile ranges (rectangular boxes), and 95%PI (whiskers). C) Cumulative incidence rate by number of yearly sexual partners and period. Bars represent mean values and whiskers represent 95%PI.

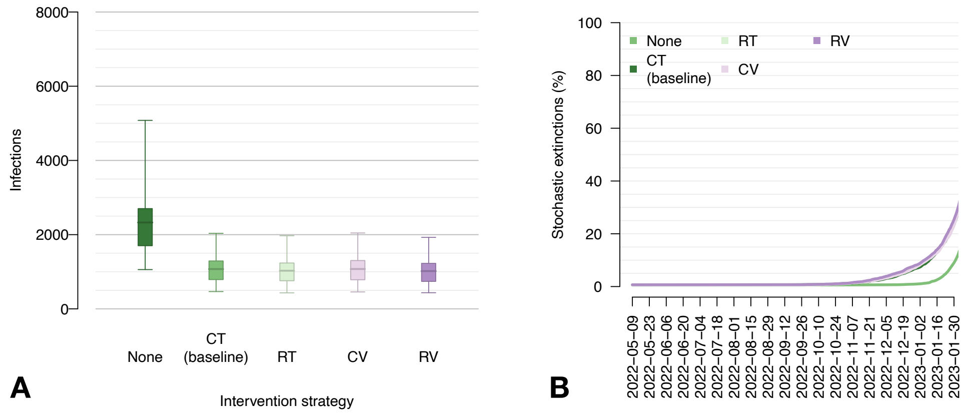

Figure S30. Alternative intervention scenarios under the assumption that clubs are attended by MSM irrespectively of their sexual activity level. A) Total number of infections across the simulation period (May 9, 2022 – February 28, 2023), by intervention. None: no active intervention; CT: simple contact tracing; RT: ring tracing; CV: vaccination of contacts; RV: ring vaccination. Boxplots represent mean values (central bars), interquartile ranges (rectangular boxes), and 95%PI (whiskers). B) Cumulative percentage of simulations that result in a stochastic extinction over time, by type of intervention. A stochastic extinction occurs when there are no more infectious or exposed individuals in the population.

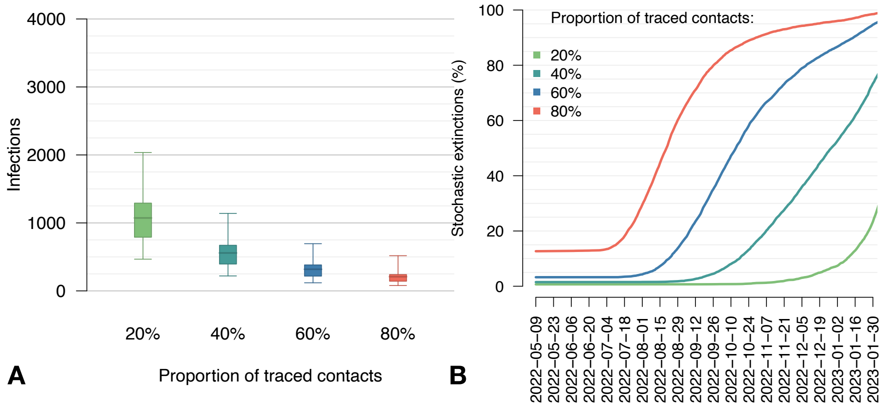

Figure S31. Alternative proportions of sexual contacts that are successfully traced under the assumption that clubs are attended by MSM irrespectively of their sexual activity level. A) Total number of infections across the simulation period (May 9, 2022 – February 28, 2023), by proportion of traced contacts. None: no active intervention; CT: simple contact tracing; RT: ring tracing; CV: vaccination of contacts; RV: ring vaccination. Boxplots represent mean values (central bars), interquartile ranges (rectangular boxes), and 95%PI (whiskers). B) Cumulative percentage of simulations that result in a stochastic extinction over time, by proportion of traced contacts. A stochastic extinction occurs when there are no more infectious or exposed individuals in the population.

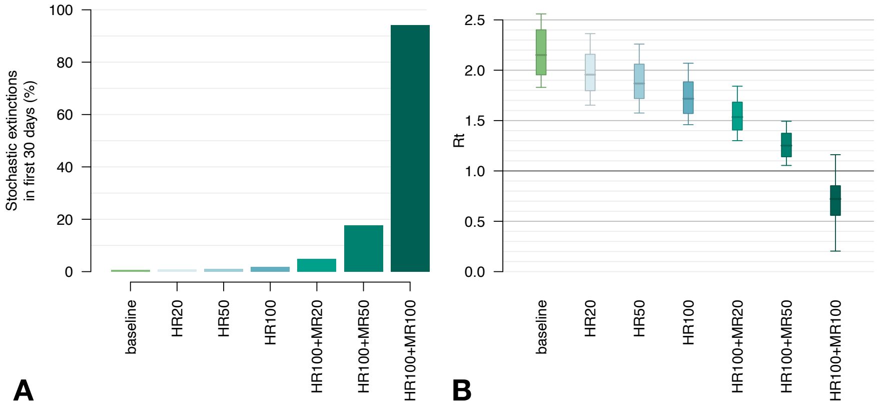

Figure S32. Effect of preventive vaccination on a potential new mpox outbreak under the assumption that clubs are attended by MSM irrespectively of their sexual activity level. A) Percentage of simulations that end up in a stochastic extinction within 30 days since the first case, by vaccination coverage. A stochastic extinction occurs when there are no more infectious or exposed individuals in the population. HR20: vaccinating 20% of high-risk (HR) MSM, i.e., those with 30 sexual partners per year or more; HR50: vaccinating 50% of HR MSM; HR100: vaccinating all HR MSM; MR20: vaccinating 20% of moderate risk (MR) MSM, i.e., those with 11 to 29 sexual partners per year; MR50: vaccinating 50% of MR MSM; MR100: vaccinating all MR MSM. Boxplots represent mean values (central bars), interquartile ranges (rectangular boxes), and 95%PI (whiskers). B) Reproduction number by vaccination coverage.

5. Supplementary references

1. Database - Eurostat. https://ec.europa.eu/eurostat/web/main/data/database.
2. Fumanelli, L., Ajelli, M., Manfredi, P., Vespignani, A. & Merler, S. Inferring the Structure of Social Contacts from Demographic Data in the Analysis of Infectious Diseases Spread. *PLOS Comput. Biol.* **8**, e1002673 (2012).
3. Sexual orientation, England and Wales - Office for National Statistics. https://www.ons.gov.uk/peoplepopulationandcommunity/culturalidentity/sexuality/bulletins/sexualorientationenglandandwales/census2021.
4. EMIS-2017 National Reports – Italy. Indagine su salute, comportamenti a rischio e bisogni di prevenzione tra le persone gay, bisessuali, ed altri MSM. https://www.emis-project.eu/emis-2017-national-reports/.
5. ARCO - Associazione Ricreativa Circoli Omosessuali. *Arco* https://www.arco.lgbt/.
6. Guzzetta, G. *et al.* Early Estimates of Monkeypox Incubation Period, Generation Time, and Reproduction Number, Italy, May–June 2022. *Emerg. Infect. Dis.* **28**, 2078–2081 (2022).
7. Brand, S. P. C. *et al.* The role of vaccination and public awareness in forecasts of Mpox incidence in the United Kingdom. *Nat. Commun.* **14**, 4100 (2023).
8. Xiridou, M. *et al.* The fading of the mpox outbreak among men who have sex with men: a mathematical modelling study. 2023.01.31.23285294 Preprint at https://doi.org/10.1101/2023.01.31.23285294 (2023).
9. Spicknall, I. H. Modeling the Impact of Sexual Networks in the Transmission of Monkeypox virus Among Gay, Bisexual, and Other Men Who Have Sex With Men — United States, 2022. *MMWR Morb. Mortal. Wkly. Rep.* **71**, (2022).
10. Suñer, C. *et al.* Viral dynamics in patients with monkeypox infection: a prospective cohort study in Spain. *Lancet Infect. Dis.* **0**, (2022).
11. Bertran, M. *et al.* Effectiveness of one dose of MVA–BN smallpox vaccine against mpox in England using the case-coverage method: an observational study. *Lancet Infect. Dis.* **0**, (2023).
12. Jezek, Z. *et al.* Serological survey for human monkeypox infections in a selected population in Zaire. *J. Trop. Med. Hyg.* **90**, 31–38 (1987).
13. Fine, P. E., Jezek, Z., Grab, B. & Dixon, H. The transmission potential of monkeypox virus in human populations. *Int. J. Epidemiol.* **17**, 643–650 (1988).

14. Overton, E. T. *et al.* Safety and Immunogenicity of Modified Vaccinia Ankara-Bavarian Nordic Smallpox Vaccine in Vaccinia-Naive and Experienced Human Immunodeficiency Virus-Infected Individuals: An Open-Label, Controlled Clinical Phase II Trial. *Open Forum Infect. Dis.* **2**, ofv040 (2015).

15. Czado, C., Gneiting, T. & Held, L. Predictive Model Assessment for Count Data. *Biometrics* **65**, 1254–1261 (2009).
