# Supplementary material for: The decline of the 2022 Italian mpox epidemic: Role of behavior changes and control strategies
Source: Nat Commun. 2024 Mar 13;15:2283. doi: 10.1038/s41467-024-46590-4 (PMC10937928; doi:10.1038/s41467-024-46590-4)
Supplement: Supplementary file 3 — Reporting Summary [file 41467_2024_46590_MOESM3_ESM.pdf]

nature portfolio

Corresponding author(s): Stefano Merler

Last updated by author(s): Feb 23, 2024

Reporting Summary

Nature Portfolio wishes to improve the reproducibility of the work that we publish. This form provides structure for consistency and transparency in reporting. For further information on Nature Portfolio policies, see our Editorial Policies and the Editorial Policy Checklist.

Statistics

For all statistical analyses, confirm that the following items are present in the figure legend, table legend, main text, or Methods section.

n/a	Confirmed	
☐	☒	The exact sample size (*n*) for each experimental group/condition, given as a discrete number and unit of measurement
☒	☐	A statement on whether measurements were taken from distinct samples or whether the same sample was measured repeatedly
☒	☐	The statistical test(s) used AND whether they are one- or two-sided<br>*Only common tests should be described solely by name; describe more complex techniques in the Methods section.*
☒	☐	A description of all covariates tested
☒	☐	A description of any assumptions or corrections, such as tests of normality and adjustment for multiple comparisons
☐	☒	A full description of the statistical parameters including central tendency (e.g. means) or other basic estimates (e.g. regression coefficient) AND variation (e.g. standard deviation) or associated estimates of uncertainty (e.g. confidence intervals)
☒	☐	For null hypothesis testing, the test statistic (e.g. *F*, *t*, *r*) with confidence intervals, effect sizes, degrees of freedom and *P* value noted<br>*Give P values as exact values whenever suitable.*
☐	☒	For Bayesian analysis, information on the choice of priors and Markov chain Monte Carlo settings
☒	☐	For hierarchical and complex designs, identification of the appropriate level for tests and full reporting of outcomes
☒	☐	Estimates of effect sizes (e.g. Cohen's *d*, Pearson's *r*), indicating how they were calculated

Our web collection on statistics for biologists contains articles on many of the points above.

Software and code

Policy information about availability of computer code

Data collection	Surveillance data were collected through PREMAL, the Italian national platform collecting data on all notifiable infectious diseases.
Data analysis	We used a custom C code for the mpox Individual Based Model. The software used in this manuscript can be accessed at: http://

For manuscripts utilizing custom algorithms or software that are central to the research but not yet described in published literature, software must be made available to editors and reviewers. We strongly encourage code deposition in a community repository (e.g. GitHub). See the Nature Portfolio guidelines for submitting code & software for further information.

Data

Policy information about availability of data

All manuscripts must include a data availability statement. This statement should provide the following information, where applicable:

- Accession codes, unique identifiers, or web links for publicly available datasets
- A description of any restrictions on data availability
- For clinical datasets or third party data, please ensure that the statement adheres to our policy

The data used in this manuscript can be accessed at: http://

Research involving human participants, their data, or biological material

Policy information about studies with human participants or human data. See also policy information about sex, gender (identity/presentation), and sexual orientation and race, ethnicity and racism.

Reporting on sex and gender	This study does not report data on sex and gender.
Reporting on race, ethnicity, or other socially relevant groupings	This study does not report data on race, ethnicity, or other socially relevant groupings.
Population characteristics	This study uses an anonymized line-list obtained during surveillance activities, with individual data on confirmed mpox cases, including age, gender, self-identification as an MSM, dates of possible exposure, date of symptom onset, date of diagnosis, epidemiological investigations, and the existence of other known cases among their contacts at the time of diagnosis. Only aggregated surveillance data on confirmed mpox cases by date of symptom onset and aggregated data on potential settings of exposure from epidemiological investigations have been used and reported in the manuscript and data repository.
Recruitment	This study reports surveillance data on confirmed mpox cases, diagnosed after spontaneous healthcare seeking or via contact tracing activities.
Ethics oversight	Ethical approval was not required for this study, as data were collected during governmental activities of infectious disease surveillance.

Note that full information on the approval of the study protocol must also be provided in the manuscript.

Field-specific reporting

Please select the one below that is the best fit for your research. If you are not sure, read the appropriate sections before making your selection.

☒ Life sciences ☐ Behavioural & social sciences ☐ Ecological, evolutionary & environmental sciences

For a reference copy of the document with all sections, see nature.com/documents/nr-reporting-summary-flat.pdf

Life sciences study design

All studies must disclose on these points even when the disclosure is negative.

Sample size	Modeling studies are not clinical trials and do not require the computation of sample sizes. The model reproduces observational data on the number of cases occurred by appropriately considering their stochastic variability. The overall number of observed cases was 921.
Data exclusions	No data were excluded from this study.
Replication	The software used in this manuscript has been run by different users on multiple computers, providing reproducible results.
Randomization	All confirmed cases were included so randomization was not relevant for this study
Blinding	All confirmed cases were included so blinding was not relevant for this study

Reporting for specific materials, systems and methods

We require information from authors about some types of materials, experimental systems and methods used in many studies. Here, indicate whether each material, system or method listed is relevant to your study. If you are not sure if a list item applies to your research, read the appropriate section before selecting a response.

Materials & experimental systems

n/a	Involved in the study	
☒	☐	Antibodies
☒	☐	Eukaryotic cell lines
☒	☐	Palaeontology and archaeology
☒	☐	Animals and other organisms
☒	☐	Clinical data
☒	☐	Dual use research of concern
☒	☐	Plants

Methods

n/a	Involved in the study	
☒	☐	ChIP-seq
☒	☐	Flow cytometry
☒	☐	MRI-based neuroimaging
